# Spontaneous Recovery of the Vestibulo-Ocular Reflex After Vestibular Neuritis; Long-Term Monitoring With the Video Head Impulse Test in a Single Patient

**DOI:** 10.3389/fneur.2020.00732

**Published:** 2020-07-28

**Authors:** Leigh Andrew McGarvie, Hamish Gavin MacDougall, Ian S. Curthoys, Gabor Michael Halmagyi

**Affiliations:** ^1^Neurology Department, Institute of Clinical Neurosciences, Royal Prince Alfred Hospital, Camperdown, NSW, Australia; ^2^Vestibular Research Laboratory, School of Psychology, The University of Sydney, Sydney, NSW, Australia

**Keywords:** vestibular neuritis, vestibulo-ocular reflex, VOR, vHIT, VOR recovery, temporal profile

## Abstract

Vestibular rehabilitation of patients in whom the level of vestibular function is continuously changing requires different strategies than in those where vestibular function rapidly becomes stable: where it recovers or where it does not and compensation is by catch-up saccades. In order to determine which of these situations apply to a particular patient, it is necessary to monitor the vestibulo-ocular reflex (VOR) gains, rather than just make a single measurement at a given time. The video Head Impulse Test (vHIT) is a simple and practical way to monitor precisely the time course and final level of VOR recovery and is useful when a patient has ongoing vestibular symptoms, such as after acute vestibular neuritis. In this study, we try to show the value of ongoing monitoring of vestibular function in a patient recovering from vestibular neuritis. Acute vestibular neuritis can impair function of any single semicircular canal (SCC). The level of impairment of each SCC, initially anywhere between 0 and 100%, can be accurately measured by the vHIT. In superior vestibular neuritis the anterior and lateral SCCs are the most affected. Unlike after surgical unilateral vestibular deafferentation, SCC function as measured by the VOR can recover spontaneously after acute vestibular neuritis. Here we report monitoring the VOR from all 6 SCCs for 500 days after the second attack in a patient with bilateral sequential vestibular neuritis. Spontaneous recovery of the VOR in response to anterior and lateral SCC impulses showed an exponential recovery with a time to reach stable levels being longer than previously considered or reported. VOR gain in response to low-velocity lateral SCC impulses recovered with a time constant of around 100 days and reached a stable level at about 200 days. However, in response to high-velocity lateral SCC and anterior SCC impulses, VOR gain recovered with a time constant of about 150 days and only reached a stable level toward the end of the 500 days monitoring period.

## Introduction

In humans and in animals, total surgical deafferentation of one labyrinth immediately produces a permanent, severe deficit of the angular vestibulo-ocular reflex (VOR) responses to rapid angular head accelerations in the off-direction of any semicircular canal (SCC) on the intact side. Catch-up, compensatory saccades substitute for the eye position error created by the VOR deficit; their cumulative magnitude is an index of the total VOR deficit during a head impulse. These are the fundamental principles underlying the video Head Impulse Test (vHIT) (1). If on the other hand the deafferentation is due to a reversible process, say, acute vestibular *neuritis*, then the deafferentation might not be permanent so that the VOR can recover, partly or fully, with or without treatment. Here we report the results of meticulous monitoring of the VOR over 500 days, with vHIT from each of the 6 SCCs of a single patient with acute vestibular neuritis and show that spontaneous recovery of SCC function can take longer than generally expected with consequences for the patient's recovery and rehabilitation. Our aim is to emphasize the ease and value of regular vHIT monitoring of the VOR during recovery from a peripheral vestibular lesion.

## Case History

**Figure 1:**
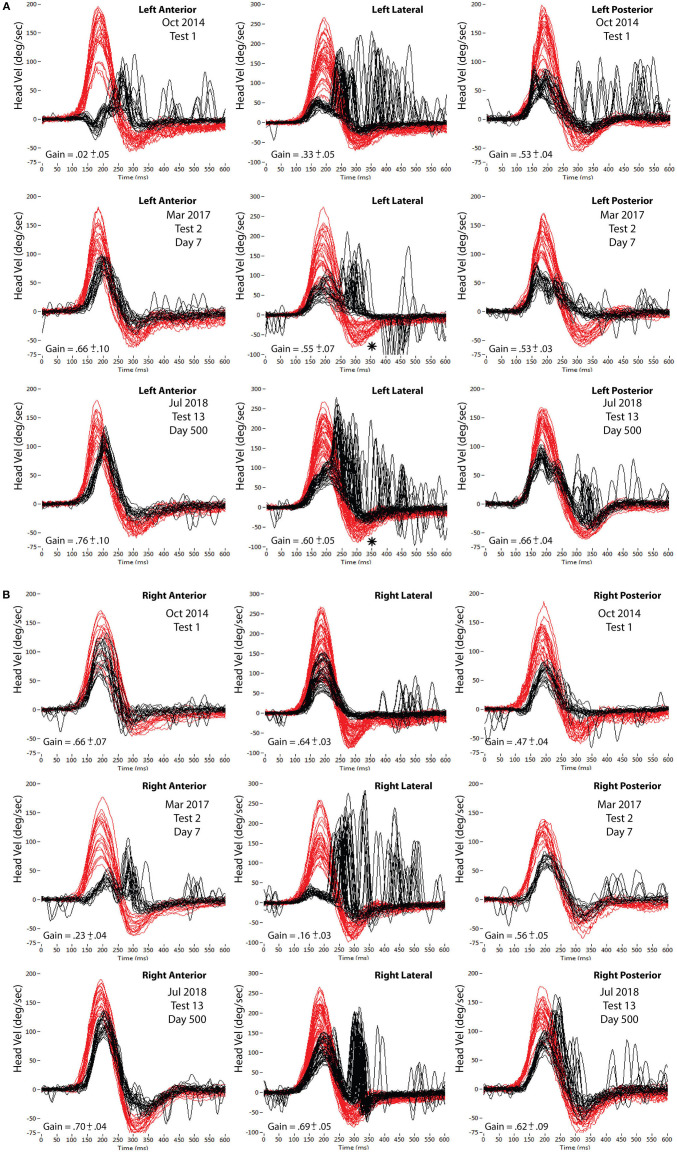
**(A)** Impulsive testing of the three left SCCs on three occasions. Top row (Test 1, Oct 2014) at presentation with left vestibular neuritis. Middle row (Test 2, May 2017) at presentation with right vestibular neuritis. Bottom row (Test 13, July 2018) last test at end of the study. Superimposed head impulses of range of head velocities (in red) with VOR eye responses (in black)—inverted. Lateral velocity scale −100 to 300; Vertical velocity scale −75 to 200. VOR gain values are average values across the full head velocity range. *marks “bounce-back” discussed in text. **(B)** Impulsive testing of the three right SCCs on three occasions. Top row (Test 1, Oct 2014), at presentation with left vestibular neuritis. Middle row (Test 2, May 2017) at presentation with right vestibular neuritis. Bottom row (Test 13, July 2018) last test at end of the study. Superimposed head impulses of range of head velocities (in red) with VOR eye responses (in black) —inverted. Lateral velocity scale −100 to 300; Vertical velocity scale −75 to 200. VOR gain values are average values across the full head velocity range.

**Figure 2:**
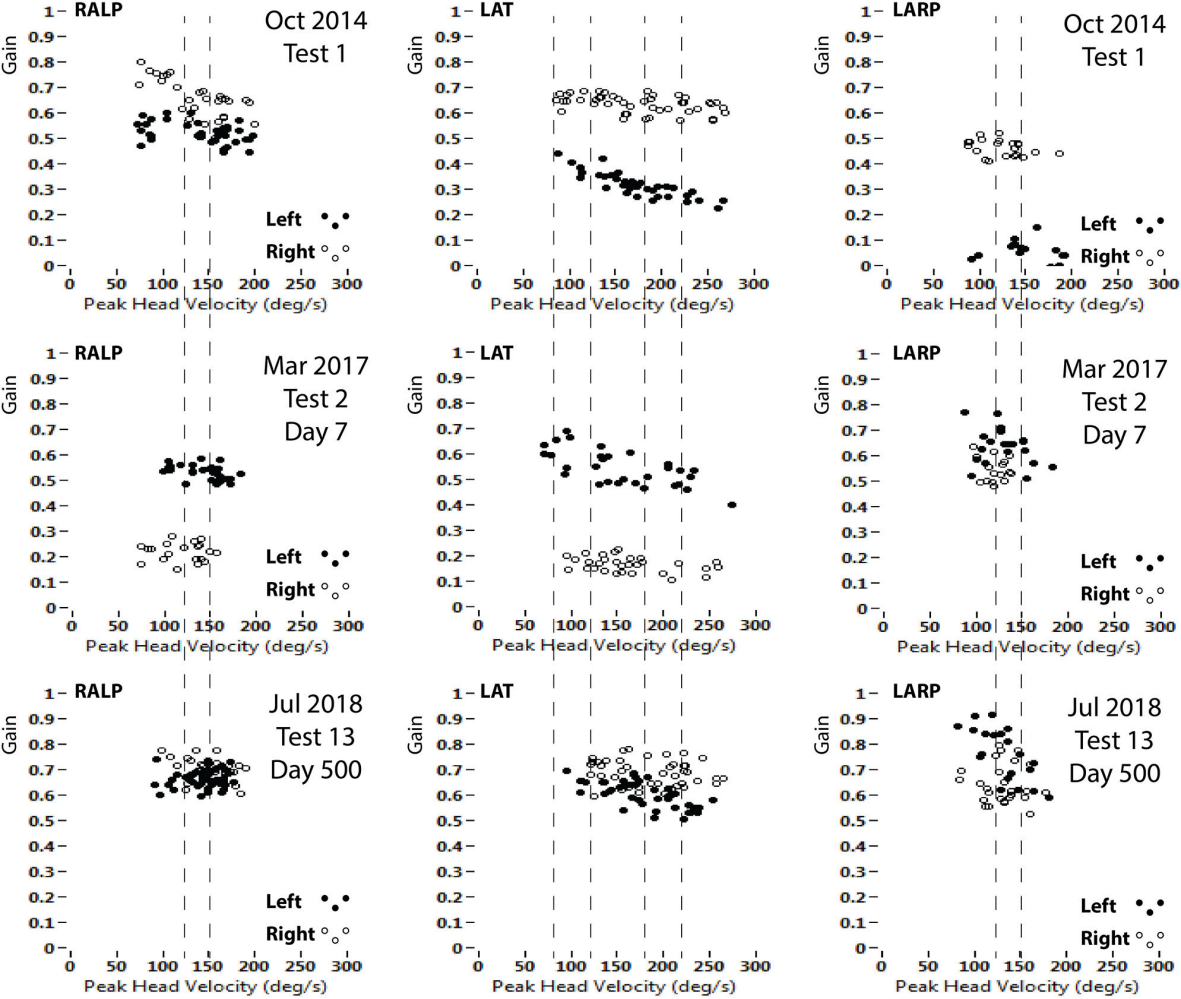
VOR gains (area under the curve of eye/head velocity) from each of the six semicircular canals. Top row (Test 1, Oct 2014) at presentation with left vestibular neuritis. Middle row (Test 2, May 2017) at presentation with right vestibular neuritis. Bottom row (Test 13, July 2018) last test at the end of the study. Left column: RALP impulses (Right anterior-Left posterior canal plane). Center column: LAT impulses (Right Lateral-Left Lateral canal plane). Right column: LARP impulses (Left anterior-Right posterior canal plane). Head impulse velocity on horizontal axes; VOR gain on vertical axes (open circles are Left; filled circles are Right). The pairs of dashed lines indicate the head velocity ranges for analysis of VOR gains in Figures 3–6.

An otherwise healthy 47 years old male presented in October 2014, 10 days after the acute onset of his first ever attack of isolated spontaneous vertigo, nausea, and vomiting. On examination he had 3°/s right-beating nystagmus suppressed by visual fixation. We diagnosed left superior vestibular neuritis. The vHIT results showed average VOR gains for the left SCCs of; lateral = 0.33; anterior = 0.02; posterior = 0.53. (Figures 1A,B, 2: top row) He also had 12° leftward deviation of the subjective visual horizontal (2). Audiogram showed normal hearing. He undertook a rehabilitation exercise program and his overall balance improved to a level that he considered fully recovered.

Two and a half years later, in March 2017, he presented a week after the onset of a 2nd acute vertigo attack. He now had 2°/s left-beating nystagmus suppressed by visual fixation. Average VOR gains now showed a right superior vestibular neuritis pattern with impaired function of the right SCCs; lateral = 0.16, anterior = 0.23, posterior = 0.56. (Figures 1A,B, 2: center row) He also had a 5° rightward deviation of the subjective visual horizontal. Cervical and ocular VEMPs were normal. After excluding Cogan's syndrome by slit lamp exam, and syphilis by negative serum TPHA (CSF was not examined), the clinical diagnosis was now *bilateral sequential vestibular neuritis* (3). As the patient was fit and healthy, we advised him to get as much outdoor activity as he could, especially in more challenging environments such as walking in the forest or on the beach, and one-on-one basketball. We emphasized that head movements in all planes during these activities should be maximized with a range of velocities. Subsequently, he reported that while vertigo and head turn oscillopsia lessened (particularly in the 1st month) they were ongoing at a low level over the period of testing, particularly when he was tired or stressed. The patient expressed interest in having the recovery of his vestibular function monitored and gave written informed consent in accordance with the Declaration of Helsinki to an ongoing vHIT protocol (X15-0266 HREC/11/RPAH/104) which was carried out during 12 tests over the next 500 days.

## Methods

All vHITs were carried out by the same right-handed operator (author LAM) using the same video goggles under the same lighting conditions. The patient was positioned 120 cm away from the target, an 8 mm diameter black dot set at eye level. VOR gain was calculated by an *area under the curve* algorithm from the start of the impulse until head velocity crossed zero (4). VOR gain is dependent upon head velocity (5), particularly when a large range of peak head velocity stimuli are delivered, as in the horizontal VOR gains shown in Figure 2. Therefore, we analyzed in detail the VOR responses to two bands of peak horizontal head velocity (80–120°/s and 180–220°/s) and to a single band of peak vertical head velocity (120–150°/s).

The data was processed by software written in LabVIEW (author HGM), which automatically detected the impulses and aligned them around peak head acceleration, into a display epoch of 600 ms. Each trace was visually checked and data displaying either artifacts or eye movements at the onset of the impulse were omitted from the final processing. VOR gain was then calculated by the software, which also processed the data into bands of peak head velocity, outputting the mean VOR gain, the standard deviation and the number of impulses within each band.

## Results

The first set of vHIT measurements for monitoring VOR gain changes were made 1 week after onset of the right vestibular neuritis (Test 2; Mar 2017).

The bins of head velocity used to compare VOR gains were: Low-velocity lateral SCC: 80–120°/s; High-velocity lateral SCC:180–220°/s.

All vertical bins used were for head velocities in the range 120–150°/s. (Figures 1A,B, 2):

The calculated VOR gains for Test 2 were:

**Right SCCs**. Low-velocity lateral SCC = 0.19 ± 0.03; high-velocity lateral SCC = 0.14 ± 0.03; anterior SCC = 0.22 ± 0.04, and posterior SCC = 0.55 ± 0.03.

**Left SCCs**. Low-velocity lateral SCC = 0.55 ± 0.02; High-velocity lateral SCC = 0.51 ± 0.02, anterior SCC = 0.67 ± 0.07, posterior SCC = 0.53 ± 0.05.

During the 12 tests over the subsequent 500 days, the right lateral and right anterior SCC VOR gains increased at an apparently exponential rate, allowing for fluctuations. To test the validity of this assumption, single exponential curves were fitted to the data (see the Supplementary Information section for fitting techniques, graphs, and equations). The results obtained from this technique were a time constant of 150 days (*R*^2^ of fit = 0.99) for the high velocity right lateral SCC impulses, a time constant of 98 days (*R*^2^ of fit = 0.997) for the low velocity right lateral SCC impulses and a time constant of 164 days (*R*^2^ of fit = 0.977) for the right anterior SCC impulses. While the fitted curves give a good estimation for the lateral data, the measured anterior SCC VOR gains show more fluctuations hence the curve fitting is less representative as shown by the lower *R*^2^ of fit value.

**Figure 3:**
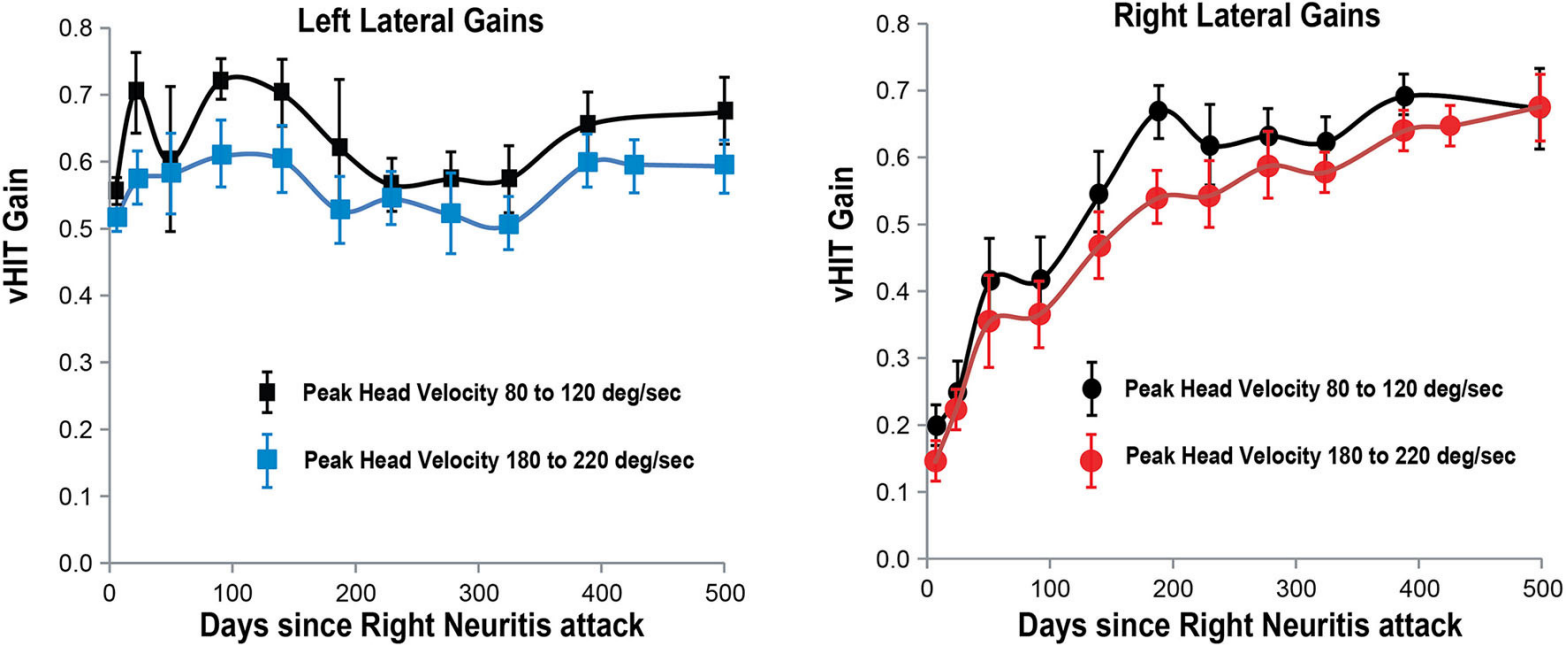
Lateral SCC VOR gain for head velocities in the range 180–220°/s and 80–120°/s (vertical axis) as a function of days (horizontal axis) after the onset of right vestibular neuritis. High-velocity right lateral SCC VOR gain in red circles; left lateral SCC gain in blue squares; Low-velocity right lateral SCC VOR gain in black circles; left lateral SCC gain in black squares. Mean and standard deviation. Gain from the left lateral SCC stays about the same over 500 days. For the right lateral SCC high-velocity impulses, it increases exponentially from 0.14 +/– 0.03 to 0.67 +/– 0.05 with a time constant of around 150 days.

**Figure 4:**
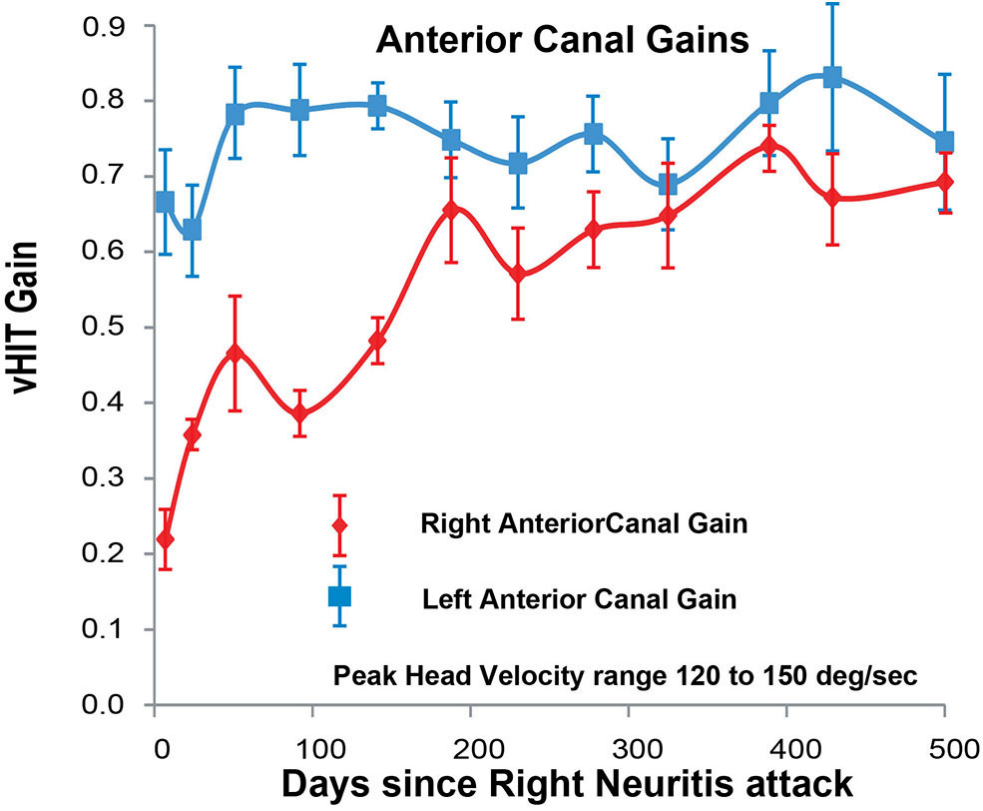
Anterior SCC VOR gain for head velocities in the range 120–150°/s (vertical axis) as a function of days (horizontal axis) after the onset of right vestibular neuritis. Right anterior SCC VOR gain in red diamonds; left anterior SCC VOR gain in blue squares. Mean and standard deviation. For the right anterior SCC VOR gain increases exponentially from 0.22 +/– 0.04 to 0.69 +/– 0.04 with a time constant of around 100–150 days estimated from 63% of the range, or 164 days calculated from an exponential curve fitted through all the points (see Supplementary Information). VOR gain from the left anterior SCC stays almost constant around an average value of 0.72.

**Figure 5:**
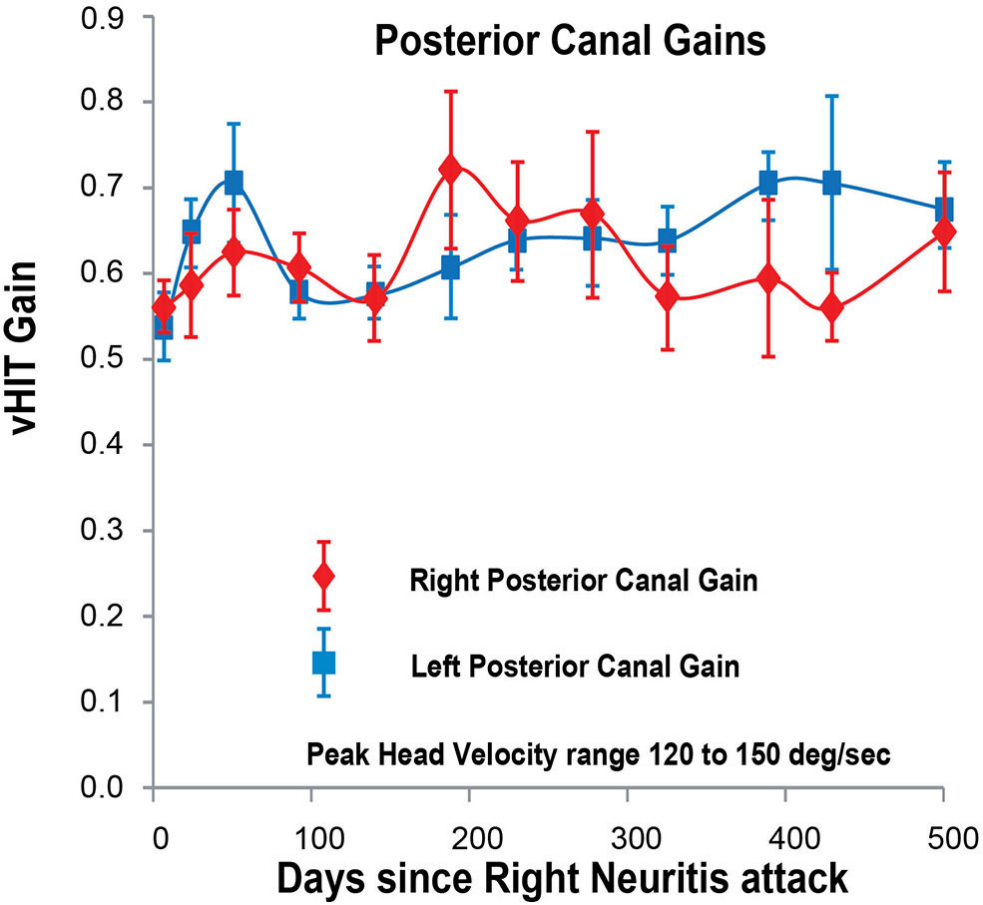
Posterior SCC VOR gain for head velocities in the range 120–150°/s (vertical axis) as a function of days (horizontal axis) after the onset of right vestibular neuritis. Right posterior SCC VOR gain in red diamonds; left posterior SCC VOR gain in blue squares. Mean and standard deviation. Both gains stay about the same over the period of observation, averaging 0.60 for the right and 0.62 for the left.

While left anterior SCC VOR gain and both left and right posterior SCC VOR gains were constant over the 500 days (Figures 3–5), right anterior SCC VOR gain increased exponentially from 0.22 to 0.69 with a temporal profile similar to that of right lateral SCC VOR gain.

Even though average left lateral SCC VOR gain remained constant over the 500 days at 0.56, the profile of its catch-up saccades changed between test 2 and test 13 (compare left lateral impulses of test 2; Figure 1A center panel, with test 13; Figure 1A; lower center panel). As average right lateral SCC VOR gain increased from 0.16 to 0.69 between tests 2 and 13 (Figure 1B), the profile of eye velocity correspondingly changes during the rightward “bounce-back” of the leftward lateral head impulse, producing a change in the subsequent catch-up saccades. The “bounce-back” (^*^ on these panels of Figure 1A) in head velocity occurs as the operator tries to stop the leftward impulse rapidly, leading to a smaller rightward head velocity before the head actually stops. Effectively, each impulse profile in any plane comprises the intended high, ipsilateral head velocity, followed by an unintended lower velocity contralateral head braking required to bring the head to a stop.

Right lateral SCC VOR gain recovered and stabilized sooner in response to low-velocity head impulses (80–120°/s), reaching a stable level at about 200 days, than in response to high-velocity head impulses (180–220°/s), which reached a stable value only after about 500 days (Figure 3). The effect of head velocity on VOR gain could not be accurately determined for the vertical SCCs as the maximum head velocity that can be delivered for the vertical SCCs is around 180–200°/s (Figure 2).

Deviation of the subjective visual horizontal at the onset of the right vestibular neuritis (Day 7) was 5.24 ± 0.45° rightward (clockwise); 20 days later it was in the normal range at 2.44 ± 0.41° and at its final value of 0.77 ± 0.36° at 50 days.

## Discussion

Immediately after total, permanent, surgical destruction, or deafferentation of an intact labyrinth, in an animal or human, there is an acute static vestibular syndrome with vertigo, nystagmus, and an ipsiversive ocular tilt reaction (6). Through brainstem compensation this syndrome always resolves, spontaneously and almost completely within a few days (7). Unilateral vestibular deafferentiation/destruction also impairs the dynamic VOR in response to rapid angular accelerations (such as head impulses) toward any of the lesioned SCCs. In contrast to the static acute vestibular syndrome, this dynamic VOR impairment is permanent (8). From these facts it follows that if after any acute unilateral vestibular impairment (e.g., after acute vestibular neuritis) there is, as shown here, VOR recovery in response to head impulses, this recovery must be due to improvement in peripheral SCC function and not to brainstem compensation.

Acute *vestibular neuritis* is more of a clinical and pathophysiological concept than a specific disease, such as say, optic neuritis. The term covers almost any attack of acute, isolated, idiopathic, unilateral impairment of peripheral vestibular function (9). While any or all of the five vestibular sensory regions might be involved, vestibular impairment is often limited to sensory regions innervated by the superior vestibular nerve: anterior SCC, lateral SCC and utricle, so this pattern is called *superior vestibular neuritis*. If impairment is confined to regions innervated by the inferior vestibular nerve (posterior SCC and saccule) this is called *inferior vestibular neuritis* (10). Some use the term *vestibular neuronitis*, (11)—implying that the lesion involves vestibular ganglion cells. If the patient also develops Benign Positional Vertigo (BPV) the term *neurolabyrinthis* (12) is used. If the other side is involved later, then this is called *bilateral sequential vestibular neuritis* (3), if at the same time it is called *acute bilateral superior branch vestibular neuropathy* (13). If hearing is also involved then the inner ear is assumed to be the site of lesion and the diagnosis becomes *labyrinthitis* (14). [Unless the patient has herpes zoster with vestibular and cochlear and nerves involved (15)]. For some or all of these reasons some prefer the simple, non-committal term, *acute unilateral peripheral vestibulopathy* (16). Here we will continue to call it “vestibular neuritis.”

The site and nature of the lesion in vestibular neuritis is uncertain. Vestibular tests, like auditory tests can lateralize a lesion but unlike auditory tests cannot localize it along the neural pathway from end-organ to brainstem nucleus. They cannot distinguish impaired vestibular function due to a disorder of vestibular hair cells from a disorder of vestibular neurons (ganglion cells, their axons, or brainstem nucleus neurons). So that while there are tests which indicate a *retro-cochlear* site of lesion for hearing loss, there are no tests to indicate *retro-labyrinthine* site of lesion for vestibular loss. Furthermore, there is no temporal bone pathology of vestibular neuritis in the acute stage; there are only a few case reports and only from temporal bones collected years after the acute event. These show loss of hair cells as well as of ganglion cells and their axons in the vestibular nerve (17).

Previous studies have shown that there usually is some spontaneous improvement of VOR gain in response to head impulses over weeks or months (18–23). In some cases there is even total recovery whereas in others there is none at all (24). If VOR recovery is incomplete then catch-up saccades, covert and overt, compensate for eye the position error produced by the deficient VOR.

**Figure 6:**
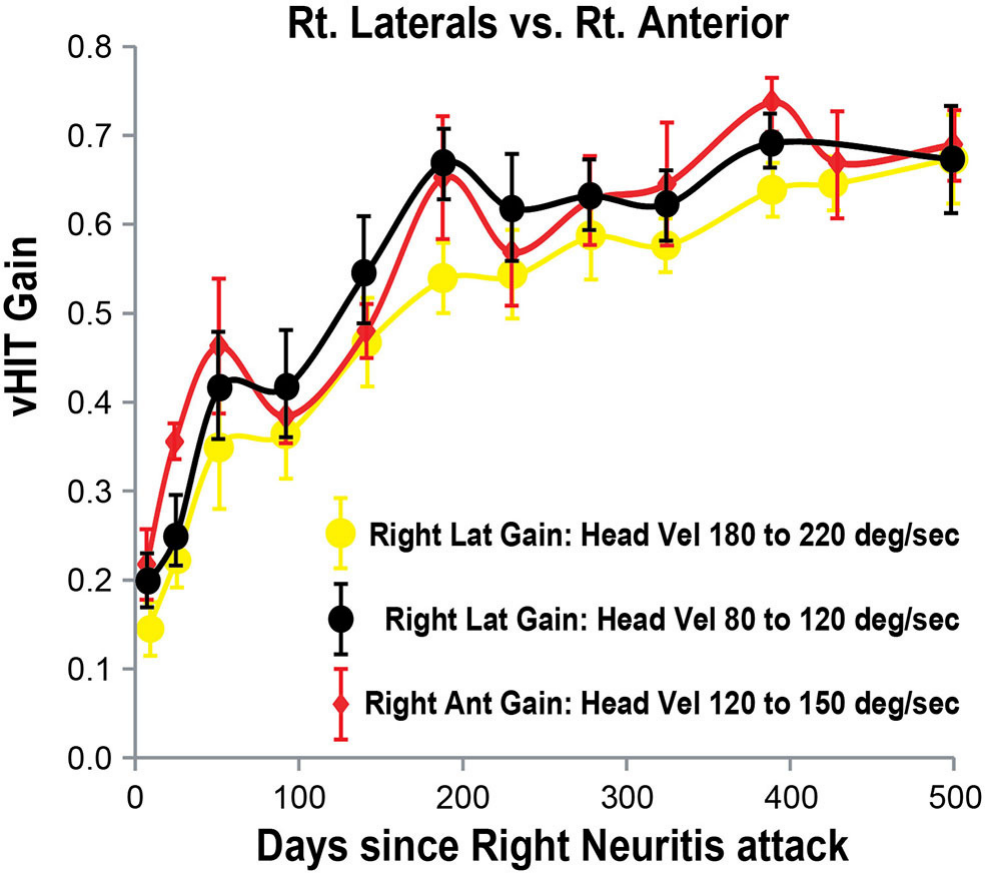
Right lateral and anterior SCC VOR gains (vertical axis) as a function of days (horizontal axis) after the onset of right vestibular neuritis. Right lateral SCC VOR gain at higher head velocity (180–220°/s) in yellow circles. Right lateral SCC VOR gain at lower head velocity (80–120°/s) in black circles. Right anterior SCC VOR gain, at 120–150°/s head velocity, shown in red diamonds. Mean and standard deviation. Note that VOR gain increase and fluctuations from the lateral SCC at lower head velocity tends to match anterior SCC VOR gain.

In our patient with acute right vestibular neuritis, right lateral SCC VOR gain in response to high-velocity head impulses recovered spontaneously, with an exponential time constant of ~150 days. The VOR gain curves for the right anterior SCC and for both low-velocity and for high-velocity responses of the right lateral SCC, when overlaid (Figure 6) show similar fluctuations over time with exponential time constants of 164, 98, and 150 days, respectively (see Supplementary Information for curve fitting equations).

During the initial steeper increase of the right SCC VOR gain over the first 3–4 weeks, the fellow left SCC VOR gains also increased, to lesser extent, and mainly at low head-velocity (Figures 3–5). So, as right lateral SCC high-velocity VOR gain increased from 0.14 to 0.35, left lateral SCC low-velocity VOR gain increased from 0.55 to 0.60. As right anterior SCC VOR gain increased from 0.22 to 0.47, left posterior SCC VOR gain increased from 0.53 to 0.70. These increases of the left SCC VOR gains in response to contralesional but ipsilateral leftward head impulses are, presumably, due to an increase in the crossed disinhibitory boost that the left vestibular nucleus type 1 excitatory neurons would normally receive from reduced activation of the right SCC afferents during leftward head impulses.

A salient point of this study is how long it can take for VOR gain to stabilize after acute vestibular neuritis. VOR gain at the higher horizontal head velocities took more than a year and a half to stabilize, so the patient was not be able to compensate effectively for the vestibular deficit during this period. Catch-up saccades are required to compensate when VOR gain is inadequate to stabilize gaze in space during head rotation. Once VOR gain has stabilized at any particular level, the system is able to “learn” the size of the saccades required to reorient gaze on the target for that given VOR level, with overt visually driven saccades initially correcting for any post-impulse gaze misalignment. However, while VOR gain is changing, either decreasing or increasing, then the size of the required saccades at any given head velocity also need to change so that the combined vestibular and visual system responses will be unable to accurately compensate until VOR gains stabilize.

In our patient right lateral SCC VOR gain increased slowly but steadily over a period of 500 days after the acute right vestibular neuritis, to a reach final level similar to left lateral SCC VOR gain (both about 0.65), with catch-up saccades correcting for the remaining deficit. In our view, the time-course of vestibular recovery we show here, more closely resembles the time-course of peripheral nerve conduction recovery from demyelination—as in acute inflammatory demyelination peripheral neuropathy (Guillain-Barre syndrome), than from axonal degeneration.

## Limitations of The Study

This study was designed to demonstrate the value of ongoing monitoring of peripheral VOR gain during a patient's recovery period, specifically demonstrating the ease of use of the vHIT and consequently does not consider caloric or rotating chair tests. As there is only one patient studied, it cannot be extended to characterize a general time course of neuritis recovery, but rather provides an example of one potential outcome.

During the 12th test (day 429), there was insufficient time available to collect the low-velocity lateral SCC impulses in the range of 80–120°/s, and so this point is not plotted on the low-velocity lateral SCC VOR gain curves.

## Conclusions

The video head impulse test enables clinicians themselves to monitor easily, accurately and regularly VOR gain from each SCC. Monitoring VOR gain could help guide rehabilitation since recovery of SCC function after acute vestibular neuritis can take longer than expected.

We emphasize that this is not meant to be a study of a particular disease. Rather it is an attempt to show how easy it is with vHIT, a method comparable in degree of difficulty to an audiogram, to monitor precisely and as often as needed, semicircular canal function over weeks, months, or years. vHIT can be used to determine whether semicircular canal function is stable or is changing, with time or with treatment. We are not specifically trying to characterize the recovery from vestibular neuritis; we are trying to show that with the correct tools, we can expand our understanding of the range of the outcomes of this and any other vestibular disorder.

## Data Availability Statement

The raw data supporting the conclusions of this article will be made available by the authors, without undue reservation.

## Ethics Statement

The studies involving human participants were reviewed and approved by Royal Prince Alfred Hospital Ethics Committee approval X15-0266 HREC/11/RPAH/104. The patients/participants provided their written informed consent to participate in this study.

## Author Contributions

LM and GH designed the study, which was carried out by LM, and wrote the paper. HM wrote the software used by LM to analyze the data. LM, HM, IC, and GH were involved in the interpretation of the data. LM prepared the figures. All authors revised the manuscript.

## Conflict of Interest

The authors declare that the research was conducted in the absence of any commercial or financial relationships that could be construed as a potential conflict of interest.
