# Supplementary material for: Spontaneous Recovery of the Vestibulo-Ocular Reflex After Vestibular Neuritis; Long-Term Monitoring With the Video Head Impulse Test in a Single Patient
Source: Front Neurol. 2020 Jul 28;11:732. doi: 10.3389/fneur.2020.00732 (PMC7399092; doi:10.3389/fneur.2020.00732)

Supplementary Information: Exponential Curve Fitting of the Data.

In the initial basic calculations, the curves were assumed to be single exponential curves and the time constants were estimated by measuring the time at which the gain curves crossed 63 % of the difference between the initial starting value and the average final value. These gave approximate time constants of about 100 days for the low velocity lateral and anterior responses, and about 150 days for the high velocity lateral response.

However, in order to improve these estimations, and to test the validity of the single exponential model, the data were fitted with single exponential curves using the Excel solver function. An equation of the form:

$$y=a-b*exp**(-c*x)$$

Where y = VOR gain, a = constant (asymptotic value of gain), b = constant, c = constant (1/time constant) and x= time in days; was fitted with initial estimations of a, b and c.

The calculated fit values of y were subtracted from the measured y values and each residual was squared. The sum of these squared errors was entered as the value for the excel solver to minimize and the output was the values of a, b and c which minimized the sum of squares of the error. The fitted curves obtained and the measured data are presented below:


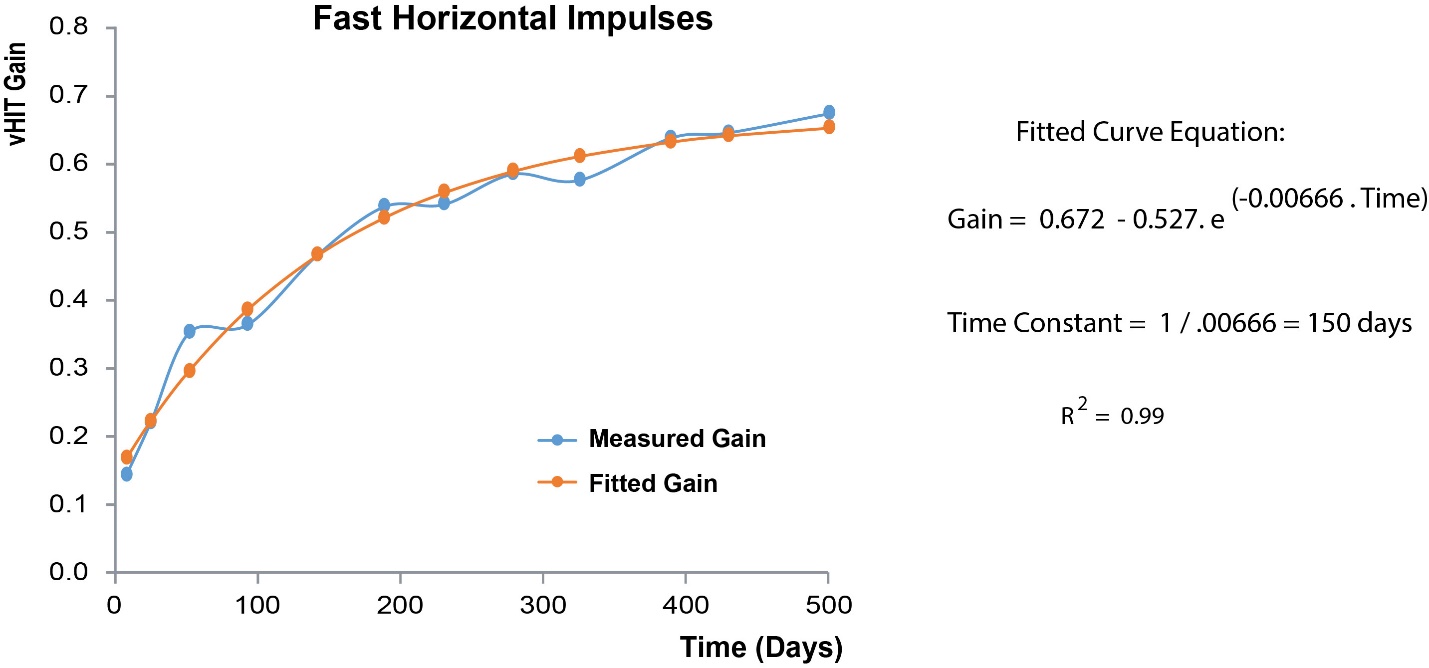


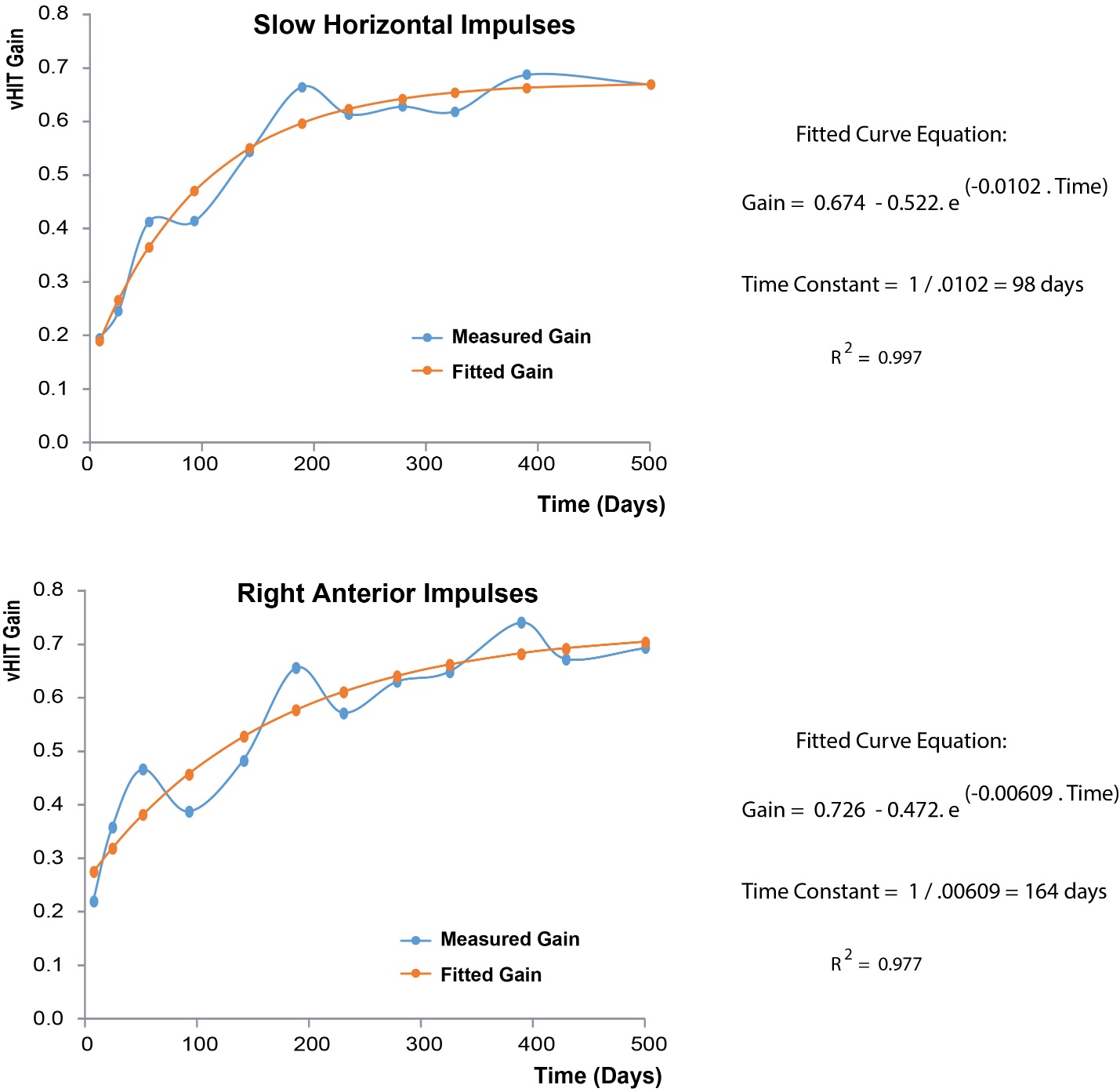

Supplement: Supplementary file 1 [file Data_Sheet_1.docx]
